# Exploring Doctors’ Willingness to Provide Online Counseling Services: The Roles of Motivations and Costs

**DOI:** 10.3390/ijerph17010110

**Published:** 2019-12-22

**Authors:** Jiahe Chen, Yi-Chen Lan, Yu-Wei Chang, Po-Ya Chang

**Affiliations:** 1 Department of Computer Science, Hong Kong Baptist University, Hong Kong, China; 2 School of Business, Western Sydney University, Sydney, NSW 2751, Australia; 3 Department of Business Management, National Taichung University of Science and Technology, Taichung City 404, Taiwan; 4 Department of Leisure Industry and Health Promotion, National Taipei University of Nursing and Health Sciences, Taipei City 112, Taiwan

**Keywords:** online health platform, eHealth, expectancy theory, BDB model, online consultation, behavioral intention

## Abstract

With the impetus of information communication technology (ICT), emerging eHealth has attracted increasing number of doctors’ participation in online health platforms, which provide various potential benefits to doctors. However, previous studies on eHealth have seldom distinguished different service modes provided by doctors. In addition, the bulk of the literature has considered doctors’ motivations based solely on online environments. To fill this gap, this study combines expectancy theory and the Bagozzi, Dholakia, and Basuroy (BDB) model to examine the relationships between anticipated outcomes, performance expectations, and effort intentions from online and offline perspectives. Doctors’ behavioral intentions are further divided into two categories: the willingness to offer free services and paid services. Using SmartPLS, this study conducts structural equation modeling (SEM) to analyze 311 sample data. The results show that extrinsic motivations (i.e., extrinsic rewards, expected relationships, and image) and intrinsic motivation (i.e., a sense of self-worth) significantly influence the desire to serve patients well, which in turn positively affects the willingness to offer free services and the willingness to offer paid services. Moreover, counseling time is confirmed as the main cost, which negatively moderates the relationships between desire and behavioral intentions. The findings provide theoretical insights for eHealth and provide practical suggestions to develop marketing strategies for online health platform providers.

## 1. Introduction

The rapid changes in information communication technology (ICT) have transformed the traditional healthcare industry. With the prevalence of the Internet, doctors can easily offer online counseling services and converse with their patients on the online health platforms anytime and anywhere. This new counseling mode, also known as eHealth, has attracted increasing attention from both doctors and patients [1,2,3,4,5,6,7,8,9]. Emerging eHealth aims to combine the features of information technology and healthcare resources to play the role of health agency in bridging public health demand and professional online counseling services [10]. eHealth provides the online platforms where doctors can share medical knowledge in virtual health communities and offer online medical consultations [1], while patients can search for health information and make an appointment for offline treatment based on online doctors’ advice [7,10].

From the perspective of doctors, online health platforms can provide doctors with various benefits pertaining to online platforms and offline hospitals. For example, by providing satisfactory online counseling services, doctors can acquire more material rewards from increased consultations online and outpatients offline and improve their professional status [1,9,11]. Under the effect of the interaction between online health platforms and offline hospitals, both the online and offline contexts should be considered when investigating doctors’ online behavioral intentions.

The above-mentioned outcomes can only be attained if doctors’ online counseling services are performed well. However, the bulk of previous studies on doctors’ participation in eHealth have only established the direct associations between motivations and behavioral intentions, but have ignored the role of individual performance expectations [1,2]. In addition, most of today’s online health platforms, such as Good Doctor, Spring Rain Doctor, and Dingxiang Doctor, provide both free and paid counseling modes [5,6,7]. However, to the best of our knowledge, no prior research on eHealth has distinguished between doctors’ behavioral intentions in relation to different charging modes, and past literature has merely investigated the relationships between motivations and single behavioral intentions [1,2,11,12]. 

To fill this gap, this study combines expectancy theory and the Bagozzi, Dholakia, and Basuroy (BDB) model to explore the motivating roles of anticipated outcomes and performance expectations in doctors’ willingness to offer free or paid online counseling services. In this study, anticipated outcomes include extrinsic rewards, expected relationships, image, and the sense of self-worth, while performance expectations are the desire to serve patients well. In addition, this study also characterizes the number of consultations and the counseling time as potential costs.

Without the active participation of doctors, online health platforms cannot play their roles in public health. Therefore, conducting an investigation to understand the motivating factors affecting doctors’ participation is very important and necessary for the long-term development of eHealth. In particular, we aim to address the following questions:

(1) How do extrinsic and intrinsic motivations affect the desire to serve patients well?
(2) How does the desire to serve patients well affect the willingness to offer free or paid online counseling services?
(3) How do the number of consultations and the counseling time moderate the relationships between desire and willingness?

## 2. Research Hypotheses

### 2.1. Anticipated Outcomes

Extrinsic rewards in this study are defined as the extent to which a doctor expects that he/she can receive extrinsic returns from both online and offline channels when providing online counseling services [12]. In the work environment, extrinsic rewards are characterized as monetary rewards and job promotion [13]. They will help motivate professionals to share knowledge based on the certain extrinsic rewards [14,15,16]. Thus, extrinsic rewards are widely validated as powerful predictors of individual behaviors and are used as attractive tactics to provoke potential participants’ enthusiasm. Similarly, in the context of eHealth, if a doctor deems that he/she can acquire benefits, such as an increased number of outpatients and online consultations and increased monetary incomes from offline hospitals and online platforms, he/she will be more willing to serve patients well to acquire these potential rewards. Therefore, this study hypothesizes the following:

**Hypothesis 1** **(H1).** *Extrinsic rewards can positively affect the desire to serve patients well.*

Expected relationships in this study are defined as the extent to which a doctor believes that he/she can foster mutual relationships with both online and offline patients when offering online counseling services [12]. Mutual relationships between individuals cannot be directly measured by material value, but they are considered extrinsic motivations [17]. Additionally, expected relationships can also be interpreted as reciprocity [13]. An individual’s initial offer to another person may establish a friendly association with each other, which in turn has a long-term effect on their reciprocal relationships. In the literature, expected relationships have also been regarded as important factors affecting individuals’ knowledge sharing behaviors and information systems (IS) adoption [15,16,18]. Similarly, in the eHealth setting, doctors’ excellent performance of online counseling services may facilitate establishing a friendly patient–doctor relationships. The close interaction between doctors and patients is important for doctors to manage their patients, so that doctors can more easily acquire patients’ trust and loyalty. Therefore, if a doctor is eager to acquire expected relationships with patients, he/she will be more willing to serve them well on online health platforms. Thus, this study hypothesizes the following:

**Hypothesis 2** **(H2).** *Expected relationships can positively affect the desire to serve patients well.*

Image in this study is defined as the extent to which a doctor’s online and offline reputation is increased through offering online counseling services [17]. In an organization, an individual cares about his/her image and tends to obtain positive appraisals and establish his/her professional status [16,17]. For example, by sharing knowledge to help colleagues, he/she can accumulate the reputation and gain the respect [19]. In eHealth, doctors’ online performance can not only be closely related to their online image, but can also influence their image in the offline hospitals. Thus, if a doctor provides a satisfactory service online, it may help his/her simultaneously gain reputation from both online and offline channels through word of mouth communication. Therefore, this study hypothesizes the following:

**Hypothesis 3** **(H3).** *Image can positively affect doctors’ desire to serve patients well.*

The sense of self-worth in this study is defined as the extent of a doctor’s positive perception based on the feeling about his/her contributions to online and offline patients through online counseling services [12]. The sense of self-worth is a purely self-motivational factor and can be also regarded as an intrinsic motivation. When knowledge contributors find that their offerings are beneficial to others and are positively evaluated, their confidence and sense of self-achievement will be increased, which in turn can also facilitate their participation in knowledge sharing [1,12,16,20]. Generally, doctors usually regard their missions as helping more patients, so they are more likely to engage in eHealth. In other words, if a doctor believes that he/she can gain a sense of self-worth that he/she values, he/she will be more likely to form a desire to serve patients well. Therefore, this study hypothesizes the following:

**Hypothesis 4** **(H4).** *A Sense of self-worth can positively affect the desire to serve patients well.*

### 2.2. Performance Expectations and Effort Intentions

In this study, performance expectations are generalized as the desire to serve patients well, while effort intentions are characterized as the willingness to offer free services and paid services. Doctors would form multiple effort intentions to achieve the performance goal of serving patients well [21]. From a doctor’s perspective in eHealth, if a doctor’s desire to serve patients well is strong, he/she will be willing to perform specific actions to attain the desire [14,22], such as offering free and paid counseling services. In the literature, performance expectations have been validated as essential factors affecting behavioral intentions [23,24]. Therefore, this study hypothesizes the following:

**Hypothesis 5** **(H5).** *The desire to serve patients well positively affects the willingness to offer free services.*

**Hypothesis 6** **(H6).** *The desire to serve patients well positively affects the willingness to offer paid services.*

### 2.3. Costs

Costs in this study involve the number of consultations and the counseling time. The former refers to the number of patients consulting with a doctor each day in eHealth, while the latter refers to the daily amount of time a doctor spends on online counseling services. Offering online counseling services is not a mandatory activity for doctors. Thus, when doctors decide to offer online counseling services in their free time, the number of consultations and extra devoted time are considered to be negative factors affecting doctors’ initiatives. The previous studies have also confirmed the negative roles of time and number. For example, time and workload were found to negatively influence the quality and quantity of knowledge contributions [15], as well as knowledge-sharing intentions [1]. Although doctors can be motivated by the aforementioned anticipated outcomes to serve patients well, they are likely to be reluctant to take further actions if they have to spend much time and effort in providing services in eHealth. Therefore, this study also regards time and number as moderators and hypothesizes the following:

**Hypothesis 7** **(H7).** *The number of consultations negatively moderates the relationship between the desire to serve patients well and the willingness to offer free services.*

**Hypothesis 8** **(H8).** *The number of consultations negatively moderates the relationship between the desire to serve patients well and the willingness to offer paid services.*

**Hypothesis 9** **(H9).** *Counseling time negatively moderates the relationship between the desire to serve patients well and the willingness to offer free services.*

**Hypothesis 10** **(H10).** *Counseling time negatively moderates the relationship between the desire to serve patients well and the willingness to offer paid services.*

**Figure 1:**
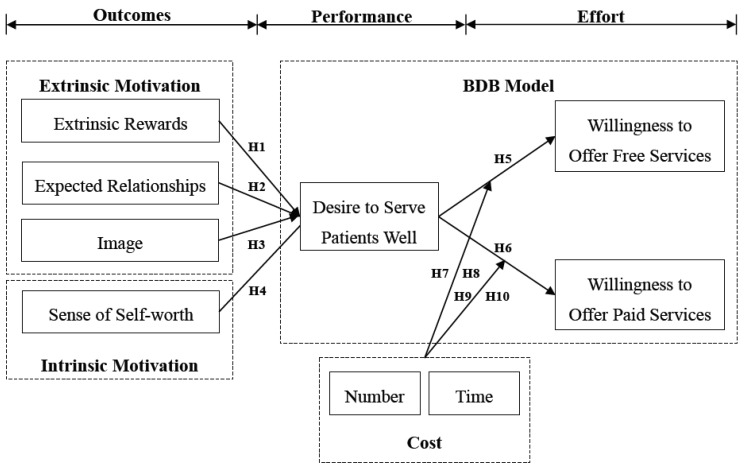
Research model. BDB, Bagozzi, Dholakia, and Basuroy.

The research model and theoretical framework are shown in Figure 1, and relevant factors are identified and summarized in Appendix A (Table A1).

## 3. Research Methodology

### 3.1. Theoretical Foundation

#### 3.1.1. Expectancy Theory

Expectancy theory has been widely applied to explain individual behavioral intentions [25,26,27] and was first proposed by Vroom [28]. Expectancy theory provides theoretical perspectives to depict and explain individual evaluations, initiatives, and decisions about the effort used to engage in a certain activity [25,29,30]. Moreover, the process of an individual’s behavioral intention can be divided into three phases: anticipated outcomes, performance expectations, and effort intentions [14]. In other words, Vroom [28] suggested that an individual will first evaluate the various attractiveness associated with achieving a certain goal. Subsequently, based on the assessment of the potential outcomes, an individual will decide to whether devote the effort to performing the feasible actions to attain the goal.

In the context of eHealth, expectancy theory is suitable for explaining how a variety of anticipated outcomes can drive doctors’ performance expectations, that is, their desire to serve patients well. The anticipated outcomes can be further categorized as extrinsic and intrinsic motivations [13,14,15]. Extrinsic motivations refer to the benefits pursued as the expected goals of organizations and individuals, such as organizational rewards, image, and reciprocity [17], while intrinsic motivations refer to the benefits that are inherently desired, such as a sense of self-worth and perceived contribution [1,17]. In this study, we consider extrinsic rewards, expected relationships, and image as the main extrinsic motivations and the sense of self-worth as the main intrinsic motivation. Doctors will systematically evaluate these potential motivations, which will lead to the formation of their desire to serve their patients well. In order to successfully achieve the goals, doctors will devote effort to performing specific actions, that is, offering free and paid counseling services. Therefore, this study used expectancy theory as a theoretical framework.

#### 3.1.2. BDB Model

The BDB model claims that an individual’s goal desire and implementation desire should precede his/her goal intention and implementation intention, respectively [14,18,21]. The desire is suggested as a necessary antecedent of behavioral intentions toward goal attainment, which is defined as the determinant of individuals’ decision-making [21]. On the basis of the BDB model, the implementation desire shapes multiple feasible implementation intentions (i.e., willingness to offer free or paid online counseling services). Therefore, this study adopted the BDB model to explain doctors’ effort intentions to take multiple feasible actions.

### 3.2. Ethics Statement

This study conducted a survey with the approval of Wenzhou People’s Hospital in Zhejiang Province. The researchers ensured that every participant understood the research objectives and procedures and acquired the informed consent from all participants before they engaged in the study. 

### 3.3. Participants and Procedures

This study introduced the Spring Rain Doctor platform for use as a reference by the respondents. Spring Rain Doctor was a popular online health platform in mainland China, which had tens of thousands of doctors and cooperated with many offline hospitals. Spring Rain Doctor also allowed registered doctors to provide free or paid counseling services. Doctors could converse with patients through the website, pictures, mobile phones, and even video format. In addition, doctors could observe timely feedback and service assessments of patients. With the help of the chief physicians of Wenzhou People’s Hospital, we distributed online questionnaires to the doctors who provided counseling services on the online health platforms and were familiar with the modes and benefits of eHealth. 

### 3.4. Measures

As shown in Appendix A, the measurement items were developed based on previous research. All the items were measured on a seven-point Likert scale, ranging from 1 “strongly disagree” to 7 “strongly agree”. For example, an intrinsic motivation (i.e., the sense of self-worth) was measured based on doctors’ perception of their contributions to online and offline patients. If 7 is chosen, it implies that the doctors have very strong perceptions of self-worth on online health platforms; if 1 is chosen, it implies the doctors hardly perceive the sense of self-worth on online health platforms. With regard to the measurement of costs, one measure item was designed for the number of consultations, ranging from 1 to 5 patients per day to more than 35 patients per day. Similarly, the counseling time was also measured by one item, ranging from less than 15 min per day to more than 3 h per day. Additionally, this study used the back-translation method to ensure the validity and consistency between the original and translated versions of the questionnaires [31]. After conducting a pilot study of 40 samples, the order, wording, and length of the items were slightly modified in the questionnaire. Besides, this study used SmartPLS 3.0 to test the structural equation model, including the measurement and structural models.

## 4. Results

### 4.1. Demographic Characteristics

A total of 600 questionnaires were distributed to target doctors. In total, 396 responses were received, with a response rate of 66%. After screening out the questionnaires containing incomplete or missing items, the number of valid questionnaires was 311 and the valid response rate was 51.8%. According to the demographics, male respondents accounted for 58.5% of the total. Most of respondents were between 36 and 45 years old, accounting for 41.2% of the total. The majority of respondents were attending physicians (37.3%) and had more than 12 years of work experience (34.4%). More demographics are detailed in Appendix B (Table A2).

### 4.2. Measurement Model

First, using confirmatory factor analysis (CFA), the measurement model was tested by content validity, convergent validity, and discriminant validity. This study reviewed and adapted the constructs and measurement items from previous studies and conducted a pilot test to ensure the content validity. In addition, we examined factor loadings, Cronbach’s α, composite reliability (CR), and average variance extracted (AVE) to evaluate the convergent validity [32]. As shown in Table 1 and Appendix A, Cronbach’s α and CRs for each construct are all higher than the acceptable threshold of 0.7, factor loadings for each item are all higher than the acceptable threshold of 0.7, and AVEs for each construct are all higher than the acceptable threshold of 0.5 [33,34]; thus, convergent validity is supported. Furthermore, we tested interconstruct correlation coefficients to measure the discriminant validity. The square roots of the AVEs for each construct in bold are higher than the other values in the corresponding columns and rows, which verifies the discriminant validity. Therefore, on the basis of the above outcomes, the measurement model is validated.

### 4.3. Structural Model and Discussion

**Figure 2:**
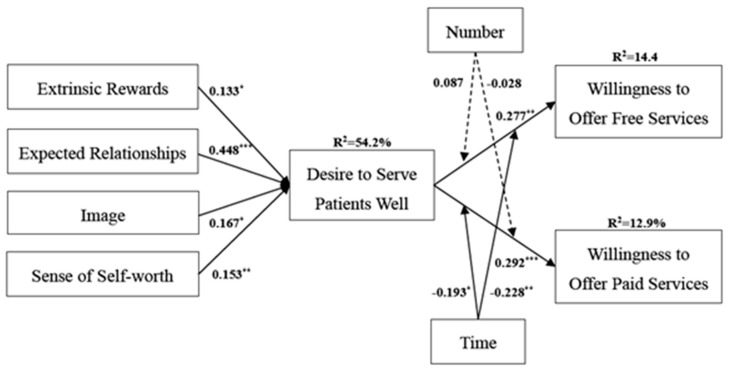
Results. * *p* < 0.05; ** *p* < 0.01; *** *p* < 0.001.

As shown in Figure 2, most of the hypotheses are validated by the data samples. Extrinsic and intrinsic motivations explain 54.2% of the variation in the desire to serve patients well. Specifically, extrinsic rewards (β = 0.133, *p* < 0.05), expected relationships (β = 0.448, *p* < 0.001), image (β = 0.167, *p* < 0.01), and the sense of self-worth (β = 0.153, *p* < 0.01) positively influence the desire to serve patients well, which support H1, H2, H3, and H4, respectively. The desire to serve patients well is found to positively influence the willingness to offer free services (β = 0.227, *p* < 0.01; R^2^ = 14.4%), as well as the willingness to offer paid services (β = 0.292, *p* < 0.001; R^2^ = 12.9%), which support H5 and H6, respectively. Thus, the results also confirm the robustness of expectancy theory. Regarding the moderators in this model, the counseling time negatively moderates the relationships between the desire to serve patients well and the willingness to offer free services (β = −0.228, *p* < 0.01) and between the desire to serve patients well and the willingness to offer paid services (β = −0.193, *p* < 0.05), thus supporting H9 and H10.

In contrast, the number of consultations has no significant moderating effect on the relationships between the desire to serve patients well and the willingness to offer free services (β = 0.087, *p* > 0.05) or between the desire to serve patients well and the willingness to offer paid services (β = −0.028, *p* > 0.05); thus, H7 and H8 are not validated. A possible explanation for this result is that the number of consultations may not accurately reflect the level of effort of the doctors compared to the counseling time. In other words, the dedication of doctors cannot be completely measured by the number of consultations in some cases. For example, in some cases, a doctor needs to spend several hours on one patient, while in other cases, a doctor may need to spend less time offering counseling services to more patients. Therefore, the role of the number of consultations in this study is insignificant.

## 5. Implications

### 5.1. Theoretical Implications

First, the previous studies have seldom simultaneously considered online and offline factors when investigating doctors’ behaviors in eHealth [1,2,11,16,35,36]. However, as there are close interactions between online health platforms and offline hospitals [4,7], it is not sufficient to focus solely on motivations provided by online channels. Therefore, this study designs the items for measuring motivations from the perspectives of both online and offline contexts. 

Second, few studies have incorporated expectancy theory and the BDB model to explain doctors’ behaviors in eHealth [1,2,9,11]. Most of the previous studies have simply drawn direct relationships between various motivations and doctors’ behavioral intentions. Therefore, this study characterizes the desire to serve patients well as a mediator connecting the motivations and behavioral intentions based on expectancy theory and justifies the formation of doctors’ two types of behavioral intentions based on the BDB model.

Third, given that online medical consultations are flexible and optional activities for doctors, anticipated costs spent in eHealth may stand out as a main concern. Thus, this study characterizes the number of consultations and the counseling time as costs of offering counseling services and tests their moderating effects on the relationships between desire and behavioral intentions.

### 5.2. Practical Implications

Because doctors’ willingness to offer online counseling services is driven by performance expectations, which are further motivated by anticipated outcomes (i.e., extrinsic rewards, expected relationships, image, and the sense of self-worth), this study also provides the following practical suggestions for online health platforms to attract doctors to provide online counseling services.

From the perspective of extrinsic rewards, online health platforms should establish and improve reward mechanisms to provide substantial rewards. For example, online health platforms can provide more subsidies and reduce platform commissions per consultation to encourage doctors to provide services. To retain the doctors, online health platforms can also formulate long-term reward programs, such as setting up the periodic rewards for continuous consultations.

From the perspective of expected relationships, online health platforms are encouraged to provide more post service functionalities, such as online patient management and group messages. Using user-friendly interfaces, doctors can easily send greetings to their patients, and track the latest health status of the patients so as to build up interactive relationships between doctors and patients.

From the perspective of image, eHealth suppliers can establish a ranking of doctors based on the patients’ overall assessment and select the top 10 doctors for month or year to recommend and display their professional status on the website homepage. In addition, online health platforms can also list the excellent counseling cases as well as doctors for patients to increase doctors’ reputation.

From the perspective of the sense of self-worth, online health platforms are recommended to establish feedback mechanisms and show patients’ real-time evaluations, such as the degree of usefulness, like button, and virtual gifts [11]. In addition, platforms can send thank you letters to thank the doctors for their dedication and exhibit the total number of patients served by the doctors and the total time invested by doctors to inspire their sense of self-achievement.

Finally, regarding the costs, online health platforms are suggested to provide caring services to alleviate the pressure on doctors and prevent overworked situations. For example, when doctors offer longtime services that exceed the upper time limit, the online health platforms should automatically send a warning message reminding them to maintain a reasonable service time.

## 6. Limitations

Nevertheless, there are still some limitations in this study that need to be explained. First, the motivations considered in this study are limited. Second, this study used cross-sectional data to conduct the investigation, which could not examine doctors’ actual behaviors. Third, the survey samples are restricted to those doctors who have participated in online health platforms, rather than inexperienced doctors.

## 7. Conclusions

The long-term good development of eHealth cannot be achieved without doctors’ active participation. Thus, it becomes very important and necessary to investigate and understand doctors’ behavioral intentions in eHealth. Considering both online and offline contexts, this study integrates expectancy theory and the BDB model to investigate the motivating roles of anticipated outcomes and performance expectations in doctors’ willingness to offer free or paid online counseling services. On the basis of the findings of the validated research hypotheses, theoretical and practical implications are also provided.

## Figures and Tables

**Table 1:** Reliability and interconstruct correlations.

Construct	Mean	S.D.	Cronbach’s α	CR	AVE	REW	REL	IMG	SSW	DES	WOF	WOP
REW	4.61	0.83	0.76	0.86	0.67	**0.82**						
REL	4.77	0.84	0.87	0.92	0.79	0.59	**0.89**					
IMG	4.77	0.89	0.78	0.87	0.70	0.63	0.69	**0.83**				
SSW	4.94	0.84	0.85	0.90	0.69	0.28	0.34	0.36	**0.83**			
DES	4.78	0.76	0.80	0.88	0.72	0.55	0.69	0.61	0.40	**0.85**		
WOF	4.14	1.11	0.92	0.95	0.86	0.20	0.25	0.15	0.06	0.26	**0.93**	
WOP	4.96	0.90	0.93	0.95	0.87	0.33	0.37	0.33	0.41	0.30	−0.12	**0.93**

Note: REW represents extrinsic rewards; REL represents expected relationships; IMG represents image; SSW represents the sense of self-worth; DES represents the desire to serve patients well; WOF represents the willingness to offer free services; WOP represents the willingness to offer paid services. The bold data values represent the square roots of the average variance extracted (AVE) of each construct. CR, composite reliability.

## Appendix A

**Table A1:** Measurement items and factor loading.

Construct	Definition and Measures	Factor Loading	Source
Extrinsic Rewards	The extent to which a doctor expects that he/she can receive extrinsic returns from both online and offline channels when providing online counseling services	-	Bock et al. [12]
	1. The income acquired from patients’ visiting my hospital would increase because of my good online counseling services	0.81	
	2. The quantity of online consultations would increase because of my good online counseling services	0.83	
	3. The quantity of patients’ visiting in my hospital would increase because of my good online counseling services	0.82	
Expected Relationships	The extent to which a doctor believes that he/she can foster mutual relationships with both online and offline patients when offering online counseling services	-	Bock et al. [12]
	1. Offering good online counseling services would strengthen the tie between me and my patients	0.88	
	2. Offering good online counseling services would draw smooth relationships with my patients	0.89	
	3. Offering good online counseling services would create strong relationships with my patients	0.90	
Image	The extent to which a doctor’s online and offline reputation is increased through offering online counseling services	-	Kankanhalli et al. [17]
	1. Offering good online counseling services would improve my image on the online health platforms and in the hospitals	0.86	
	2. Offering good online counseling services would improve others’ recognition of me	0.87	
	3. Offering good online counseling services, the people I work with respect me	0.77	
Sense of Self-worth	The extent of a doctor’s positive perception based on the feeling about his/her contribution to online and offline patients through online counseling services	-	Bock et al. [12]
	1. Offering good online counseling services would help my patients solve their health problems	0.81	
	2. Offering good online counseling services would improve my service quality	0.88	
	3. Offering good online counseling services would improve inquiry processes	0.80	
	4. Offering good online counseling services would create chances to solve patients’ health problems.	0.82	
Desire to Serve Patients Well	The strength of a doctor’s desire to serve patients well	-	Bagozzi et al. [21]
	1. I want to serve my patients well	0.80	
	2. My overall wish is to serve my patients well	0.88	
	3. I feel an urge or need to serve my patients well	0.85	
Willingness to Offer Free Services	The strength of a doctor’s willingness to offer free services	-	Perugini and Bagozzi [37]
	1. I am planning to offer free online counseling services	0.90	
	2. I will spend effort to offer free online counseling services	0.94	
	3. I intend to offer free online counseling services	0.93	
Willingness to Offer Paid Services	The strength of a doctor’s willingness to offer paid services	-	Perugini and Bagozzi [37]
	1. I am planning to offer paid online counseling services	0.92	
	2. I will spend effort to offer paid online counseling services	0.95	
	3. I intend to offer paid online counseling services	0.93	

## Appendix B

**Table A2:** Demographic statistics.

Measure	Item	Frequency	Percentage	Measure	Item	Frequency	Percentage
Gender	Male	182	58.5	Department	Internal medicine	96	30.9
	Female	129	41.5		Stomatology	59	19
Age	18–25 years	10	3.2		Traditional Chinese medicine	42	13.5
	26–35 years	117	37.6		Bone surgery	31	10
	36–45 years	128	41.2		Gynecology and obstetrics	25	8
	46–55 years	47	15.1		Surgery	21	6.7
	56–65 years	8	2.6		Pediatrics	9	2.9
	>65 years	1	0.3		Ophthalmology	5	1.6
Title	Assistant Doctor	12	3.9		Oncology	4	1.3
	Resident Doctor	68	21.9		Others	19	6.1
	Attending Physician	116	37.3	Hospital	Hospital of Zhejiang University	87	28.0
	Associate Chief Physician	77	24.8		Zhejiang Provincial Hospital of Traditional Chinese Medicine	66	21.2
	Chief Physician	25	8		Tongde Hospital of Zhejiang Province	51	16.4
	Others	13	4.2		Wenzhou People’s Hospital	38	12.2
Career age	<1 year	32	10.3		Quzhou Hospital	30	9.7
	1–2 years	42	13.5		Ningbo Hospital	29	9.3
	3–4 years	38	12.2		Hospital of Wenzhou Medical University	10	3.2
	5–6 years	26	8.4				
	7–8 years	23	7.4				
	9–10 years	26	8.4				
	11–12 years	17	5.5				
	>12 years	107	34.4				

Note: the total number of respondents is 311.

## References

[1] Yan Z., Wang T., Chen Y., Zhang H. (2016). Knowledge sharing in online health communities: A social exchange theory perspective. Inf. Manag..

[2] Zhang X., Liu S., Chen X., Gong Y. (2017). Social capital, motivations, and knowledge sharing intention in health Q & A communities. Manag. Decis..

[3] Sims J.M. (2018). Communities of practice: Telemedicine and online medical communities. Technol. Forecast. Soc. Chang..

[4] Chang Y.W., Hsu P.Y., Wang Y., Chang P.Y. (2019). Integration of online and offline health services: The role of doctor-patient online interaction. Patient Educ. Couns..

[5] Yang H., Zhang X. (2019). Investigating the Effect of Paid and Free Feedback About Physicians’ Telemedicine Services on Patients’ and Physicians’ Behaviors: Panel Data Analysis. J. Med. Internet Res..

[6] Tang Y., Yang Y.T., Shao Y.F. (2019). Acceptance of Online Medical Websites: An Empirical Study in China. Int. J. Environ. Res. Public Health.

[7] Le W., Chang P.Y., Chang Y.W., Chen J. (2019). Why Do Patients Move from Online Health Platforms to Hospitals? The Perspectives of Fairness Theory and Brand Extension Theory. Int. J. Environ. Res. Public Health.

[8] Deng Z., Liu S. (2017). Understanding consumer health information-seeking behavior from the perspective of the risk perception attitude framework and social support in mobile social media websites. Int. J. Med. Inform..

[9] Guo S., Guo X., Fang Y., Vogel D. (2017). How doctors gain social and economic returns in online health-care communities: A professional capital perspective. J. Manag. Inf. Syst..

[10] Oh H., Rizo C., Enkin M., Jadad A. (2005). What is eHealth (3): A systematic review of published definitions. J. Med. Internet Res..

[11] Liu J., Bian Y., Ye Q., Jing D. (2019). Free for Caring? The Effect of Offering Free Online Medical-Consulting Services on Physician Performance in e-Health Care. Telemed. E Health.

[12] Bock G.W., Zmud R.W., Kim Y.G., Lee J.N. (2005). Behavioral intention formation in knowledge sharing: Examining the roles of extrinsic motivators, social-psychological factors, and organizational climate. MIS Q..

[13] Bock G.W., Kim Y.G. (2002). Breaking the myths of rewards: An exploratory study of attitudes about knowledge sharing. Inf. Resour. Manag. J..

[14] Chang Y.W., Hsu P.Y., Wu Z.Y. (2015). Exploring managers’ intention to use business intelligence: The role of motivations. Behav. Inf. Technol..

[15] Sedighi M., van Splunter S., Brazier F., van Beers C., Lukosch S. (2016). Exploration of multi-layered knowledge sharing participation: The roles of perceived benefits and costs. J. Knowl. Manag..

[16] Huang Q., Davison R.M., Gu J. (2008). Impact of personal and cultural factors on knowledge sharing in China. Asia Pac. J. Manag..

[17] Kankanhalli A., Tan B.C., Wei K.K. (2005). Contributing knowledge to electronic knowledge repositories: An empirical investigation. MIS Q..

[18] Tsai M.T., Chang H.C., Cheng N.C., Lien C.C. (2013). Understanding IT professionals’ knowledge sharing intention through KMS: A social exchange perspective. Qual. Quant..

[19] Lu X., Zhou H., Chen S. (2019). Facilitate knowledge sharing by leading ethically: The role of organizational concern and impression management climate. J. Bus. Psychol..

[20] Ding G., Liu H., Huang Q., Gu J. (2017). Moderating effects of guanxi and face on the relationship between psychological motivation and knowledge-sharing in China. J. Knowl. Manag..

[21] Bagozzi R.P., Dholakia U.M., Basuroy S. (2003). How effortful decisions get enacted: The motivating role of decision processes, desires, and anticipated emotions. J. Behav. Decis. Mak..

[22] Dholakia U.M., Bagozzi R.P., Gopinath M. (2007). How formulating implementation plans and remembering past actions facilitate the enactment of effortful decisions. J. Behav. Decis. Mak..

[23] Puspitasari I., Firdauzy A. (2019). Characterizing Consumer Behavior in Leveraging Social Media for E-Patient and Health-Related Activities. Int. J. Environ. Res. Public Health.

[24] Wang M.Y., Zhang P.Z., Zhou C.Y., Lai N.Y. (2019). Effect of Emotion, Expectation, and Privacy on Purchase Intention in WeChat Health Product Consumption: The Mediating Role of Trust. Int. J. Environ. Res. Public Health.

[25] Snead K.C., Harrell A.M. (1994). An application of expectancy theory to explain a manager’s intention to use a decision support system. Decis. Sci..

[26] Lin C.P. (2017). Modeling corporate citizenship and turnover intention: Social identity and expectancy theories. Rev. Manag. Sci..

[27] Rasch R.H., Tosi H.L. (1992). Factors affecting software developers’ performance: An integrated approach. MIS Q..

[28] Vroom V.H. (1964). Work and Motivation.

[29] Chiang C.F., Jang S.S. (2008). An expectancy theory model for hotel employee motivation. Int. J. Hosp. Manag..

[30] Reinharth L., Wahba M.A. (1975). Expectancy theory as a predictor of work motivation, effort expenditure, and job performance. Acad. Manag. J..

[31] Mullen M.R. (1995). Diagnosing measurement equivalence in cross-national research. J. Int. Bus. Stud..

[32] Fornell C., Larcker D.F. (1981). Evaluating structural equation models with unobservable variables and measurement error. J. Mark. Res..

[33] Hair J.F., Black W.C., Babin B.J., Anderson R.E., Tatham R.L. (1998). Multivariate Data Analysis.

[34] Nunnally J.C. (1978). Psychometric Methods.

[35] Cao W., Zhang X., Xu K., Wang Y. (2016). Modeling online health information-seeking behavior in China: The roles of source characteristics, reward assessment, and internet self-efficacy. Health Commun..

[36] Zhou J., Fan T. (2019). Understanding the factors influencing patient E-health literacy in online health communities (OHCs): A social cognitive theory perspective. Int. J. Environ. Res. Public Health.

[37] Perugini M., Bagozzi R.P. (2001). The role of desires and anticipated emotions in goal-directed behaviours: Broadening and deepening the theory of planned behaviour. Br. J. Soc. Psychol..

